# Supplementary material for: Lagged Association of Ambient Outdoor Air Pollutants with Asthma-Related Emergency Department Visits within the Pittsburgh Region
Source: Int J Environ Res Public Health. 2020 Nov 20;17(22):8619. doi: 10.3390/ijerph17228619 (PMC7699695; doi:10.3390/ijerph17228619)
Supplement: Supplementary file 1 [file ijerph-17-08619-s001.pdf]

International Journal of
*Environmental Research and Public Health*

**Table S1.** Children/Teens Age 5–17: Asthma ED Visits, for $PM_{2.5}$ (per 10 μg/m³). Single Pollutant, Single day lags and average lag.

| Parameter | Estimate | Std Error | Chi-Square | *p*-Value | Chi-Square Ratio | 95-Confidence Intervals | |
|---|---|---|---|---|---|---|---|
| Lag 0 | | | | | | | |
| $PM_{2.5}$_10μg | −0.15351 | 0.07907 | 3.7692 | 0.0522 | 0.858 | 0.735 | 1.001 |
| AvgTemp | 0.00632 | 0.00486 | 1.6929 | 0.1932 | 1.006 | 0.997 | 1.016 |
| Lag 1 | | | | | | | |
| $PM_{2.5}$_10μg | −0.06593 | 0.07801 | 0.7143 | 0.3980 | 0.936 | 0.803 | 1.091 |
| AvgTemp | 0.00939 | 0.00482 | 3.7993 | 0.0513 | 1.009 | 1.000 | 1.019 |
| Lag 2 | | | | | | | |
| $PM_{2.5}$_10μg | −0.01152 | 0.07831 | 0.0216 | 0.8831 | 0.989 | 0.848 | 1.153 |
| AvgTemp | 0.00297 | 0.00486 | 0.3741 | 0.5408 | 1.003 | 0.993 | 1.013 |
| Lag 3 | | | | | | | |
| $PM_{2.5}$_10μg | −0.01554 | 0.07864 | 0.0390 | 0.8434 | 0.985 | 0.844 | 1.149 |
| AvgTemp | 0.00442 | 0.00486 | 0.8289 | 0.3626 | 1.004 | 0.995 | 1.014 |
| Lag 4 | | | | | | | |
| $PM_{2.5}$_10μg | **0.05658** | 0.08091 | 0.4891 | 0.4843 | 1.058 | 0.903 | 1.240 |
| AvgTemp | 0.00249 | 0.00499 | 0.2484 | 0.6182 | 1.002 | 0.993 | 1.012 |
| Lag 5 | | | | | | | |
| $PM_{2.5}$_10μg | 0.07370 | 0.08001 | 0.8484 | 0.3570 | 1.076 | 0.920 | 1.259 |
| AvgTemp | −0.00064 | 0.00505 | 0.0165 | 0.8977 | 0.999 | 0.990 | 1.009 |
| Lag 0–5 | | | | | | | |
| $PM_{2.5}$_10μg | −0.04453 | 0.12565 | 0.1256 | 0.7230 | 0.956 | 0.748 | 1.224 |
| AvgTemp | 0.00850 | 0.00686 | 1.5345 | 0.2154 | 1.009 | 0.995 | 1.022 |

Data were adjusted for apparent temperature (at same lag) analyses with a 7-day washout and 28-day referent periods. AvgTemp = Average temperature; Std Error = Standard Error.

**Table S2.** Children/Teens Age 5–17: Asthma ED Visits for Ozone (per 10 ppb). Single Pollutant, Single day lags and average lag.

| Parameter | Estimate | Std Error | Chi-Square | *p*-Value | Chi-Square Ratio | 95-Confidence Intervals | |
|---|---|---|---|---|---|---|---|
| Lag 0 | | | | | | | |
| $O_3$_10ppb | -0.00022 | 0.04200 | 0.0000 | 0.9958 | 1.000 | 0.921 | 1.086 |
| AvgTemp | 0.00389 | 0.00496 | 0.6167 | 0.4323 | 1.004 | 0.994 | 1.014 |
| Lag 1 | | | | | | | |
| $O_3$_10ppb | 0.09963 | 0.04240 | 5.5230 | 0.0188 | 1.105 | 1.017 | 1.200 |
| AvgTemp | 0.00455 | 0.00493 | 0.8507 | 0.3564 | 1.005 | 0.995 | 1.014 |
| Lag 2 | | | | | | | |
| $O_3$_10ppb | 0.01652 | 0.04155 | 0.1581 | 0.6909 | 1.017 | 0.937 | 1.103 |
| AvgTemp | 0.00211 | 0.00493 | 0.1830 | 0.6688 | 1.002 | 0.992 | 1.012 |
| Lag 3 | | | | | | | |
| $O_3$_10ppb | 0.02213 | 0.04150 | 0.2843 | 0.5939 | 1.022 | 0.943 | 1.109 |
| AvgTemp | 0.00331 | 0.00491 | 0.4528 | 0.5010 | 1.003 | 0.994 | 1.013 |
| Lag 4 | | | | | | | |
| $O_3$_10ppb | 0.04718 | 0.04227 | 1.2459 | 0.2643 | 1.048 | 0.965 | 1.139 |
| AvgTemp | 0.00182 | 0.00499 | 0.1336 | 0.7147 | 1.002 | 0.992 | 1.012 |
| Lag 5 | | | | | | | |
| $O_3$_10ppb | -0.02610 | 0.04141 | 0.3973 | 0.5285 | 0.974 | 0.898 | 1.057 |
| AvgTemp | 0.00179 | 0.00507 | 0.1249 | 0.7237 | 1.002 | 0.992 | 1.012 |
| Lag 0-5 | | | | | | | |
| $O_3$_10ppb | 0.07201 | 0.06932 | 1.0789 | 0.2989 | 1.075 | 0.938 | 1.231 |
| AvgTemp | 0.00517 | 0.00711 | 0.5295 | 0.4668 | 1.005 | 0.991 | 1.019 |

Data were adjusted for apparent temperature (at same lag) analyses with a 7-day washout and 28-day referent periods. *Abbreviation Definitions: AvgTemp = Average temperature; Std Error=Standard Error*

**Table S3.** Children/Teens Age 5–17: Asthma ED Visits for $PM_{2.5}$ (per 10 μg/m$^3$) and Ozone (per 10 ppb). Two Pollutants, Single day lags and average lag.

| Parameter | Estimate | Std Error | Chi-Square | P-value | Chi-Square Ratio | 95-Confidence Intervals | |
|---|---|---|---|---|---|---|---|
| Lag 0 | | | | | | | |
| PM 2.5_10μg | -0.16130 | 0.08112 | 3.9540 | 0.0468 | 0.851 | 0.726 | 0.998 |
| $O_3$_10ppb | 0.01891 | 0.04337 | 0.1901 | 0.6628 | 1.019 | 0.936 | 1.110 |
| AvgTemp | 0.00575 | 0.00503 | 1.3045 | 0.2534 | 1.006 | 0.996 | 1.016 |
| Lag 1 | | | | | | | |
| PM 2.5_10μg | -0.11943 | 0.08064 | 2.1937 | 0.1386 | 0.887 | 0.758 | 1.039 |
| $O_3$_10ppb | 0.11657 | 0.04410 | 6.9866 | 0.0082 | 1.124 | 1.031 | 1.225 |
| AvgTemp | 0.00585 | 0.00500 | 1.3700 | 0.2418 | 1.006 | 0.996 | 1.016 |
| Lag 2 | | | | | | | |
| PM 2.5_10μg | -0.01931 | 0.08038 | 0.0577 | 0.8101 | 0.981 | 0.838 | 1.148 |
| $O_3$_10ppb | 0.01879 | 0.04269 | 0.1937 | 0.6599 | 1.019 | 0.937 | 1.108 |
| AvgTemp | 0.00237 | 0.00505 | 0.2207 | 0.6385 | 1.002 | 0.993 | 1.012 |
| Lag 3 | | | | | | | |
| PM 2.5_10μg | -0.02641 | 0.08076 | 0.1070 | 0.7436 | 0.974 | 0.831 | 1.141 |
| $O_3$_10ppb | 0.02534 | 0.04271 | 0.3520 | 0.5530 | 1.026 | 0.943 | 1.115 |
| AvgTemp | 0.00366 | 0.00503 | 0.5297 | 0.4667 | 1.004 | 0.994 | 1.014 |
| Lag 4 | | | | | | | |
| PM 2.5_10μg | 0.03845 | 0.08296 | 0.2148 | 0.6431 | 1.039 | 0.883 | 1.223 |
| $O_3$_10ppb | 0.04266 | 0.04331 | 0.9703 | 0.3246 | 1.044 | 0.959 | 1.136 |
| AvgTemp | 0.00122 | 0.00516 | 0.0556 | 0.8136 | 1.001 | 0.991 | 1.011 |
| Lag 5 | | | | | | | |
| PM 2.5_10μg | 0.09023 | 0.08232 | 1.2013 | 0.2731 | 1.094 | 0.931 | 1.286 |
| $O_3$_10ppb | -0.03665 | 0.04233 | 0.7496 | 0.3866 | 0.964 | 0.887 | 1.047 |
| AvgTemp | 0.00047 | 0.00521 | 0.0084 | 0.9269 | 1.000 | 0.990 | 1.011 |
| Lag 0-5 | | | | | | | |
| PM 2.5_10μg | -0.09659 | 0.13249 | 0.5316 | 0.4659 | 0.908 | 0.700 | 1.177 |
| $O_3$_10ppb | 0.08952 | 0.07351 | 1.4830 | 0.2233 | 1.094 | 0.947 | 1.263 |
| AvgTemp | 0.00592 | 0.00718 | 0.6788 | 0.4100 | 1.006 | 0.992 | 1.020 |

Data were adjusted for apparent temperature (at same lag) analyses with a 7-day washout and 28-day referent periods. *Abbreviation Definitions: AvgTemp = Average temperature; Std Error=Standard Error.*

**Table S4.** Children/Teens Age 5-17: Asthma ED Visits for Carbon Monoxide (CO) (per 10 ppm). Single Pollutant, Single day lags and average lag.

| Parameter | Estimate | Std Error | Chi-Square | *p*-Value | Chi-Square Ratio | 95-Confidence Intervals | |
|---|---|---|---|---|---|---|---|
| **Lag 0** | | | | | | | |
| CO_ppm | -0.15182 | 0.17157 | 0.783 | 0.3762 | 0.859 | 0.614 | 1.203 |
| AvgTemp | 0.00496 | 0.00485 | 1.0453 | 0.3066 | 1.005 | 0.995 | 1.015 |
| Lag 1 | | | | | | | |
| CO_ppm | -0.21203 | 0.1649 | 1.6533 | 0.1985 | 0.809 | 0.586 | 1.118 |
| AvgTemp | 0.00988 | 0.00481 | 4.2115 | 0.0401 | 1.01 | 1 | 1.02 |
| Lag 2 | | | | | | | |
| CO_ppm | -0.29423 | 0.17175 | 2.9349 | 0.0867 | 0.745 | 0.532 | 1.043 |
| AvgTemp | 0.00486 | 0.00481 | 1.0221 | 0.312 | 1.005 | 0.995 | 1.014 |
| Lag 3 | | | | | | | |
| CO_ppm | -0.19924 | 0.16565 | 1.4467 | 0.2291 | 0.819 | 0.592 | 1.134 |
| AvgTemp | 0.00554 | 0.00479 | 1.3345 | 0.248 | 1.006 | 0.996 | 1.015 |
| Lag 4 | | | | | | | |
| CO_ppm | -0.07304 | 0.16261 | 0.2017 | 0.6533 | 0.93 | 0.676 | 1.278 |
| AvgTemp | 0.00419 | 0.00487 | 0.7414 | 0.3892 | 1.004 | 0.995 | 1.014 |
| Lag 5 | | | | | | | |
| CO_ppm | -0.12825 | 0.1598 | 0.6441 | 0.4222 | 0.88 | 0.643 | 1.203 |
| AvgTemp | 0.00152 | 0.00489 | 0.0967 | 0.7558 | 1.002 | 0.992 | 1.011 |
| Lag 0–5 | | | | | | | |
| CO_ppm | -0.4145 | 0.26956 | 2.3644 | 0.1241 | 0.661 | 0.39 | 1.121 |
| AvgTemp | 0.00941 | 0.00671 | 1.9697 | 0.1605 | 1.009 | 0.996 | 1.023 |

Data were adjusted for apparent temperature (at same lag) analyses with a 7-day washout and 28-day referent periods. *Abbreviation Definitions: AvgTemp = Average temperature; Std Error=Standard Error.*

**Table S5.** Children/Teens Age 5–17: Asthma ED Visits for $NO_2$ (per 10 ppb). Single Pollutant, Single day lags and average lag.

| Parameter | Estimate | Std Error | Chi-Square | *p*-Value | Chi-Square Ratio | 95-Confidence Intervals | |
|---|---|---|---|---|---|---|---|
| **Lag 0** | | | | | | | |
| NO2_10ppb | -0.05374 | 0.04799 | 1.2542 | 0.2628 | 0.948 | 0.863 | 1.041 |
| AvgTemp | 0.00501 | 0.00481 | 1.0863 | 0.2973 | 1.005 | 0.996 | 1.015 |
| Lag 1 | | | | | | | |
| NO2_10ppb | -0.02798 | 0.0483 | 0.3356 | 0.5624 | 0.972 | 0.885 | 1.069 |
| AvgTemp | 0.00887 | 0.00475 | 3.4873 | 0.0618 | 1.009 | 1 | 1.018 |
| Lag 2 | | | | | | | |
| NO2_10ppb | -0.00601 | 0.04736 | 0.0161 | 0.8991 | 0.994 | 0.906 | 1.091 |
| AvgTemp | 0.00288 | 0.00474 | 0.3692 | 0.5435 | 1.003 | 0.994 | 1.012 |
| Lag 3 | | | | | | | |
| NO2_10ppb | -0.02201 | 0.04755 | 0.2141 | 0.6435 | 0.978 | 0.891 | 1.074 |
| AvgTemp | 0.00456 | 0.00474 | 0.9264 | 0.3358 | 1.005 | 0.995 | 1.014 |
| Lag 4 | | | | | | | |
| NO2_10ppb | 0.03801 | 0.04836 | 0.6178 | 0.4319 | 1.039 | 0.945 | 1.142 |
| AvgTemp | 0.00281 | 0.00483 | 0.339 | 0.5604 | 1.003 | 0.993 | 1.012 |
| Lag 5 | | | | | | | |
| NO2_10ppb | 0.01623 | 0.04838 | 0.1126 | 0.7372 | 1.016 | 0.924 | 1.117 |
| AvgTemp | 0.00044 | 0.0049 | 0.0081 | 0.9284 | 1 | 0.991 | 1.01 |
| Lag 0–5 | | | | | | | |
| NO2_10ppb | -0.01663 | 0.08187 | 0.0412 | 0.8391 | 0.984 | 0.838 | 1.155 |
| AvgTemp | 0.00804 | 0.00668 | 1.4485 | 0.2288 | 1.008 | 0.995 | 1.021 |

Data were adjusted for apparent temperature (at same lag) analyses with a 7-day washout and 28-day referent periods. *Abbreviation Definitions: AvgTemp = Average temperature; Std Error=Standard Error.*

**Table S6.** Children/Teens Age 5–17: Asthma ED Visits for $SO_2$ (per 10 ppb). Single Pollutant, Single day lags and average lag.

| Parameter | Estimate | Std Error | Chi-Square | *p*-Value | Chi-Square Ratio | 95-Confidence Intervals | |
|---|---|---|---|---|---|---|---|
| Lag 0 | | | | | | | |
| SO2_10ppb | -0.02384 | 0.04292 | 0.3085 | 0.5786 | 0.976 | 0.898 | 1.062 |
| AvgTemp | 0.00399 | 0.0047 | 0.7183 | 0.3967 | 1.004 | 0.995 | 1.013 |
| Lag 1 | | | | | | | |
| SO2_10ppb | -0.02135 | 0.04121 | 0.2685 | 0.6044 | 0.979 | 0.903 | 1.061 |
| AvgTemp | 0.00845 | 0.00466 | 3.2845 | 0.0699 | 1.008 | 0.999 | 1.018 |
| Lag 2 | | | | | | | |
| SO2_10ppb | 0.03405 | 0.03945 | 0.7449 | 0.3881 | 1.035 | 0.958 | 1.118 |
| AvgTemp | 0.00264 | 0.00465 | 0.3222 | 0.5703 | 1.003 | 0.994 | 1.012 |
| Lag 3 | | | | | | | |
| SO2_10ppb | 0.03605 | 0.04052 | 0.7913 | 0.3737 | 1.037 | 0.958 | 1.122 |
| AvgTemp | 0.00399 | 0.00466 | 0.7334 | 0.3918 | 1.004 | 0.995 | 1.013 |
| Lag 4 | | | | | | | |
| SO2_10ppb | -0.03651 | 0.04481 | 0.6638 | 0.4152 | 0.964 | 0.883 | 1.053 |
| AvgTemp | 0.00391 | 0.00473 | 0.6831 | 0.4085 | 1.004 | 0.995 | 1.013 |
| Lag 5 | | | | | | | |
| SO2_10ppb | 0.00796 | 0.04304 | 0.0342 | 0.8533 | 1.008 | 0.926 | 1.097 |
| AvgTemp | 0.0007 | 0.00482 | 0.0212 | 0.8844 | 1.001 | 0.991 | 1.01 |
| Lag 6 | | | | | | | |
| SO2_10ppb | 0.00564 | 0.08212 | 0.0047 | 0.9452 | 1.006 | 0.856 | 1.181 |
| AvgTemp | 0.00785 | 0.00662 | 1.4034 | 0.2362 | 1.008 | 0.995 | 1.021 |

Data were adjusted for apparent temperature (at same lag) analyses with a 7-day washout and 28-day referent periods. *Abbreviation Definitions: AvgTemp = Average temperature; Std Error=Standard Error.*

**Table S7.** Children/Teens Age 5-17: Asthma ED Visits for PM2.5 (per 10 μg/m³), Ozone (per 10 ppb), CO (ppm), NO2 (per 10 ppb), SO2 (per 10 ppb). Multiple Pollutant models, Single day lags and average lag.

| Parameter | Estimate | Std Error | Chi-Square | *p*-Value | Chi-Square Ratio | 95-Confidence Intervals | |
|---|---|---|---|---|---|---|---|
| Lag 0 | | | | | | | |
| PM 2.5_10μg | -0.15374 | 0.09685 | 2.5198 | 0.1124 | 0.857 | 0.709 | 1.037 |
| O3_10ppb | 0.02007 | 0.04376 | 0.2103 | 0.6465 | 1.02 | 0.936 | 1.112 |
| NO2_10ppb | -0.00874 | 0.06113 | 0.0205 | 0.8863 | 0.991 | 0.879 | 1.117 |
| SO2_10ppb | -0.00984 | 0.04365 | 0.0508 | 0.8217 | 0.99 | 0.909 | 1.079 |
| CO_ppm | 0.01393 | 0.2072 | 0.0045 | 0.9464 | 1.014 | 0.676 | 1.522 |
| AvgTemp | 0.00571 | 0.00511 | 1.2504 | 0.2635 | 1.006 | 0.996 | 1.016 |
| Lag 1 | | | | | | | |
| PM 2.5_10μg | -0.09026 | 0.09486 | 0.9054 | 0.3413 | 0.914 | 0.759 | 1.1 |
| O3_10ppb | 0.11326 | 0.04447 | 6.4854 | 0.0109 | 1.12 | 1.026 | 1.222 |
| NO2_10ppb | 0.00478 | 0.06123 | 0.0061 | 0.9377 | 1.005 | 0.891 | 1.133 |
| SO2_10ppb | -0.01675 | 0.04192 | 0.1597 | 0.6894 | 0.983 | 0.906 | 1.068 |
| CO_ppm | -0.14172 | 0.19506 | 0.5279 | 0.4675 | 0.868 | 0.592 | 1.272 |
| AvgTemp | 0.00653 | 0.00508 | 1.6524 | 0.1986 | 1.007 | 0.997 | 1.017 |
| Lag 2 | | | | | | | |
| PM 2.5_10μg | 0.02553 | 0.09711 | 0.0691 | 0.7926 | 1.026 | 0.848 | 1.241 |
| O3_10ppb | 0.00819 | 0.0432 | 0.0359 | 0.8496 | 1.008 | 0.926 | 1.097 |
| NO2_10ppb | 0.03892 | 0.06044 | 0.4148 | 0.5196 | 1.04 | 0.924 | 1.17 |
| SO2_10ppb | 0.03843 | 0.04007 | 0.9199 | 0.3375 | 1.039 | 0.961 | 1.124 |
| CO_ppm | -0.41287 | 0.21113 | 3.8242 | 0.0505 | 0.662 | 0.438 | 1.001 |
| AvgTemp | 0.00404 | 0.00513 | 0.6199 | 0.4311 | 1.004 | 0.994 | 1.014 |
| Lag 3 | | | | | | | |
| PM 2.5_10μg | 0.01468 | 0.09777 | 0.0225 | 0.8807 | 1.015 | 0.838 | 1.229 |
| O3_10ppb | 0.01931 | 0.04314 | 0.2005 | 0.6544 | 1.02 | 0.937 | 1.109 |
| NO2_10ppb | -0.00249 | 0.05998 | 0.0017 | 0.9669 | 0.998 | 0.887 | 1.122 |
| SO2_10ppb | 0.04162 | 0.04119 | 1.0208 | 0.3123 | 1.042 | 0.962 | 1.13 |
| CO_ppm | -0.23171 | 0.20048 | 1.3358 | 0.2478 | 0.793 | 0.535 | 1.175 |
| AvgTemp | 0.00464 | 0.00509 | 0.8303 | 0.3622 | 1.005 | 0.995 | 1.015 |
| Lag 4 | | | | | | | |
| PM 2.5_10μg | 0.0473 | 0.09885 | 0.229 | 0.6323 | 1.048 | 0.864 | 1.273 |
| O3_10ppb | 0.03848 | 0.04364 | 0.7774 | 0.3779 | 1.039 | 0.954 | 1.132 |
| NO2_10ppb | 0.0542 | 0.06079 | 0.7948 | 0.3727 | 1.056 | 0.937 | 1.189 |
| SO2_10ppb | -0.04513 | 0.04617 | 0.9556 | 0.3283 | 0.956 | 0.873 | 1.046 |
| CO_ppm | -0.18486 | 0.1969 | 0.8814 | 0.3478 | 0.831 | 0.565 | 1.223 |
| AvgTemp | 0.00176 | 0.0052 | 0.1143 | 0.7353 | 1.002 | 0.992 | 1.012 |
| Lag 5 | | | | | | | |
| PM 2.5_10μg | 0.1359 | 0.09834 | 1.9098 | 0.167 | 1.146 | 0.945 | 1.389 |
| O3_10ppb | -0.04685 | 0.04295 | 1.1898 | 0.2754 | 0.954 | 0.877 | 1.038 |
| NO2_10ppb | 0.02737 | 0.06059 | 0.204 | 0.6515 | 1.028 | 0.913 | 1.157 |
| SO2_10ppb | 0.00456 | 0.04382 | 0.0108 | 0.9171 | 1.005 | 0.922 | 1.095 |
| CO_ppm | -0.29678 | 0.19516 | 2.3125 | 0.1283 | 0.743 | 0.507 | 1.09 |
| AvgTemp | 0.00117 | 0.00524 | 0.0497 | 0.8236 | 1.001 | 0.991 | 1.011 |

| | | | | | | | |
|---|---|---|---|---|---|---|---|
| Lag 0-5 | | | | | | | |
| PM 2.5_10μg | -0.05342 | 0.16391 | 0.1062 | 0.7445 | 0.948 | 0.688 | 1.307 |
| O3_10ppb | 0.07167 | 0.07456 | 0.9239 | 0.3364 | 1.074 | 0.928 | 1.243 |
| NO2_10ppb | 0.08093 | 0.11367 | 0.5069 | 0.4765 | 1.084 | 0.868 | 1.355 |
| SO2_10ppb | 0.02648 | 0.08488 | 0.0973 | 0.755 | 1.027 | 0.869 | 1.213 |
| CO_ppm | -0.54617 | 0.34314 | 2.5334 | 0.1115 | 0.579 | 0.296 | 1.135 |
| AvgTemp | 0.00709 | 0.00724 | 0.9596 | 0.3273 | 1.007 | 0.993 | 1.022 |

Data were adjusted for apparent temperature (at same lag) analyses with a 7-day washout and 28-day referent periods. *Abbreviation Definitions: AvgTemp = Average temperature; Std Error=Standard Error.*

**Table S8.** Adults 18+: Asthma ED Visits for $PM_{2.5}$ (per 10 μg/m$^3$). Single Pollutant, Single day lags and average lag.

| Parameter | Estimate | Std Error | Chi-Square | *p*-Value | Chi-Square Ratio | 95-Confidence Intervals | |
|---|---|---|---|---|---|---|---|
| Lag 0 | | | | | | | |
| PM2.5_10μg | 0.00225 | 0.02926 | 0.0059 | 0.9387 | 1.002 | 0.946 | 1.061 |
| AvgTemp | 0.0025 | 0.00184 | 1.8602 | 0.1726 | 1.003 | 0.999 | 1.006 |
| Lag 1 | | | | | | | |
| PM2.5_10μg | -0.00056 | 0.02919 | 0.0004 | 0.9846 | 0.999 | 0.944 | 1.058 |
| AvgTemp | 0.00246 | 0.00185 | 1.7602 | 0.1846 | 1.002 | 0.999 | 1.006 |
| Lag 2 | | | | | | | |
| PM2.5_10μg | -0.01998 | 0.02949 | 0.4591 | 0.4981 | 0.98 | 0.925 | 1.039 |
| AvgTemp | 0.0024 | 0.00185 | 1.6773 | 0.1953 | 1.002 | 0.999 | 1.006 |
| Lag 3 | | | | | | | |
| PM 2.5_10μg | -0.00769 | 0.02956 | 0.0677 | 0.7948 | 0.992 | 0.936 | 1.052 |
| AvgTemp | 0.00353 | 0.00185 | 3.6423 | 0.0563 | 1.004 | 1 | 1.007 |
| Lag 4 | | | | | | | |
| PM2.5_10μg | 0.00947 | 0.03002 | 0.0995 | 0.7524 | 1.01 | 0.952 | 1.071 |
| AvgTemp | 0.00076 | 0.00188 | 0.1672 | 0.6826 | 1.001 | 0.997 | 1.004 |
| Lag 5 | | | | | | | |
| PM2.5_10μg | 0.03075 | 0.03016 | 1.0393 | 0.308 | 1.031 | 0.972 | 1.094 |
| AvgTemp_ | -0.00148 | 0.00191 | 0.601 | 0.4382 | 0.999 | 0.995 | 1.002 |
| Lag 0-5 | | | | | | | |
| PM2.5_10μg | 0.0094 | 0.0481 | 0.0382 | 0.8451 | 1.009 | 0.919 | 1.109 |
| AvgTemp | 0.00335 | 0.0026 | 1.6641 | 0.1971 | 1.003 | 0.998 | 1.008 |

Data were adjusted for apparent temperature (at same lag) analyses with a 7-day washout and 28-day referent periods. *Abbreviation Definitions: AvgTemp = Average temperature; Std Error=Standard Error.*

**Table S9.** Adults 18+: Asthma ED Visits for Ozone (per 10 ppb). Single Pollutant, Single day lags and average lag.

| Parameter | Estimate | Std Error | Chi-Square | *p*-value | Chi-Square Ratio | 95-Confidence Intervals | |
|---|---|---|---|---|---|---|---|
| Lag 0 | | | | | | | |
| O3_10ppb | -0.03208 | 0.01624 | 3.9023 | 0.0482 | 0.968 | 0.938 | 1 |
| AvgTemp | 0.0037 | 0.00187 | 3.9303 | 0.0474 | 1.004 | 1 | 1.007 |
| Lag 1 | | | | | | | |
| O3_10ppb | -0.03221 | 0.01623 | 3.9384 | 0.0472 | 0.968 | 0.938 | 1 |
| AvgTemp | 0.00359 | 0.00188 | 3.6649 | 0.0556 | 1.004 | 1 | 1.007 |
| Lag 2 | | | | | | | |
| O3_10ppb | -0.04174 | 0.0162 | 6.6368 | 0.01 | 0.959 | 0.929 | 0.99 |
| AvgTemp | 0.00359 | 0.00188 | 3.6439 | 0.0563 | 1.004 | 1 | 1.007 |
| Lag 3 | | | | | | | |
| O3_10ppb | -0.03376 | 0.01613 | 4.3812 | 0.0363 | 0.967 | 0.937 | 0.998 |
| AvgTemp | 0.00464 | 0.00188 | 6.1001 | 0.0135 | 1.005 | 1.001 | 1.008 |
| Lag 4 | | | | | | | |
| O3_10ppb | -0.03166 | 0.01602 | 3.9072 | 0.0481 | 0.969 | 0.939 | 1 |
| AvgTemp | 0.00209 | 0.00189 | 1.2239 | 0.2686 | 1.002 | 0.998 | 1.006 |
| Lag | | | | | | | |
| O3_10ppb | -0.0188 | 0.01601 | 1.3791 | 0.2403 | 0.981 | 0.951 | 1.013 |
| AvgTemp | -0.00019 | 0.00193 | 0.0103 | 0.919 | 1 | 0.996 | 1.004 |
| Lag 5 | | | | | | | |
| O3_10ppb | -0.08902 | 0.0272 | 10.7116 | 0.0011 | 0.915 | 0.867 | 0.965 |
| AvgTemp | 0.00653 | 0.00268 | 5.9361 | 0.0148 | 1.007 | 1.001 | 1.012 |

Data were adjusted for apparent temperature (at same lag) analyses with a 7-day washout and 28-day referent periods. *Abbreviation Definitions: AvgTemp = Average temperature; Std Error=Standard Error.*

**Table S10.** Adults 18+: Asthma ED Visits for PM2.5 (per 10 μg/m$^3$) and Ozone (per 10 ppb). Two Pollutants, Single day lags and average lag.

| Parameter | Estimate | Std Error | Chi-Square | *p*-value | Chi-Square Ratio | 95-Confidence Intervals | |
|---|---|---|---|---|---|---|---|
| Lag 0 | | | | | | | |
| PM2.5_10μg | 0.01687 | 0.0301 | 0.3143 | 0.5751 | 1.017 | 0.959 | 1.079 |
| O3_10ppb | -0.03427 | 0.01668 | 4.2232 | 0.0399 | 0.966 | 0.935 | 0.998 |
| AvgTemp | 0.0035 | 0.0019 | 3.3991 | 0.0652 | 1.004 | 1 | 1.007 |
| Lag 1 | | | | | | | |
| PM2.5_10μg | 0.014 | 0.03003 | 0.2172 | 0.6412 | 1.014 | 0.956 | 1.076 |
| O3_10ppb | -0.03404 | 0.01668 | 4.1667 | 0.0412 | 0.967 | 0.935 | 0.999 |
| AvgTemp | 0.00342 | 0.00191 | 3.2013 | 0.0736 | 1.003 | 1 | 1.007 |
| Lag 2 | | | | | | | |
| PM2.5_10μg | -0.00141 | 0.03041 | 0.0022 | 0.963 | 0.999 | 0.941 | 1.06 |
| O3_10ppb | -0.04155 | 0.01669 | 6.1959 | 0.0128 | 0.959 | 0.928 | 0.991 |
| AvgTemp | 0.00361 | 0.00192 | 3.5457 | 0.0597 | 1.004 | 1 | 1.007 |
| Lag 3 | | | | | | | |
| PM2.5_10μg | 0.00681 | 0.03034 | 0.0503 | 0.8225 | 1.007 | 0.949 | 1.069 |
| O3_10ppb | -0.03459 | 0.01655 | 4.3715 | 0.0365 | 0.966 | 0.935 | 0.998 |
| AvgTemp | 0.00456 | 0.00191 | 5.6744 | 0.0172 | 1.005 | 1.001 | 1.008 |
| Lag 4 | | | | | | | |
| PM2.5_10μg | 0.02347 | 0.03075 | 0.583 | 0.4451 | 1.024 | 0.964 | 1.087 |
| O3_10ppb | -0.03436 | 0.01637 | 4.4072 | 0.0358 | 0.966 | 0.936 | 0.998 |
| AvgTemp | 0.00176 | 0.00194 | 0.8284 | 0.3627 | 1.002 | 0.998 | 1.006 |
| Lag 5 | | | | | | | |
| PM2.5_10μg | 0.04042 | 0.03092 | 1.7093 | 0.1911 | 1.041 | 0.98 | 1.106 |
| O3_10ppb | -0.02345 | 0.01636 | 2.0555 | 0.1517 | 0.977 | 0.946 | 1.009 |
| AvgTemp | -0.00075 | 0.00197 | 0.1463 | 0.7021 | 0.999 | 0.995 | 1.003 |
| Lag 0-5 | | | | | | | |
| PM2.5_10μg | 0.06431 | 0.05075 | 1.606 | 0.2051 | 1.066 | 0.965 | 1.178 |
| O3_10ppb | -0.1 | 0.02851 | 12.3004 | 0.0005 | 0.905 | 0.856 | 0.957 |
| AvgTemp | 0.00603 | 0.00271 | 4.9599 | 0.0259 | 1.006 | 1.001 | 1.011 |

Data were adjusted for apparent temperature (at same lag) analyses with a 7-day washout and 28-day referent periods. *Abbreviation Definitions: AvgTemp = Average temperature; Std Error=Standard Error.*

**Table S11.** Adults 18+: Asthma ED Visits for Carbon Monoxide (per 10 ppm). Single Pollutant, Single day lags and average lag.

| Parameter | Estimate | Std Error | Chi-Square | *p*-Value | Chi-Square Ratio | 95-Confidence Intervals | |
|---|---|---|---|---|---|---|---|
| Lag 0-5 | | | | | | | |
| CO_ppm | 0.08524 | 0.0638 | 1.7849 | 0.1816 | 1.089 | 0.961 | 1.234 |
| AvgTemp | 0.0019 | 0.00184 | 1.0715 | 0.3006 | 1.002 | 0.998 | 1.006 |
| Lag 1 | | | | | | | |
| CO_ppm | 0.06997 | 0.06324 | 1.2241 | 0.2686 | 1.072 | 0.947 | 1.214 |
| AvgTemp | 0.00195 | 0.00184 | 1.1213 | 0.2896 | 1.002 | 0.998 | 1.006 |
| Lag 2 | | | | | | | |
| CO_ppm | 0.03378 | 0.06376 | 0.2806 | 0.5963 | 1.034 | 0.913 | 1.172 |
| AvgTemp | 0.00182 | 0.00184 | 0.9807 | 0.322 | 1.002 | 0.998 | 1.005 |
| Lag 3 | | | | | | | |
| CO_ppm | 0.09172 | 0.06325 | 2.103 | 0.147 | 1.096 | 0.968 | 1.241 |
| AvgTemp | 0.00279 | 0.00183 | 2.326 | 0.1272 | 1.003 | 0.999 | 1.006 |
| Lag 4 | | | | | | | |
| CO_ppm | 0.0162 | 0.06277 | 0.0666 | 0.7963 | 1.016 | 0.899 | 1.149 |
| AvgTemp | 0.000833 | 0.00185 | 0.2032 | 0.6522 | 1.001 | 0.997 | 1.004 |
| Lag 5 | | | | | | | |
| CO_ppm | 0.12878 | 0.06224 | 4.2815 | 0.0385 | 1.137 | 1.007 | 1.285 |
| AvgTemp_ | -0.00173 | 0.00187 | 0.8604 | 0.3536 | 0.998 | 0.995 | 1.002 |
| Lag 0-5 | | | | | | | |
| CO_ppm | 0.20214 | 0.10229 | 3.905 | 0.0481 | 1.224 | 1.002 | 1.496 |
| AvgTemp_ | 0.00261 | 0.00255 | 1.0472 | 0.3062 | 1.003 | 0.998 | 1.008 |

Data were adjusted for apparent temperature (at same lag) analyses with a 7-day washout and 28-day referent periods. *Abbreviation Definitions: AvgTemp = Average temperature; Std Error=Standard Error.*

**Table S12.** Adults 18+: Asthma ED Visits for NO2 (per 10 ppb). Single Pollutant, Single day lags and average lag.

| Parameter | Estimate | Std Error | Chi-Square | *p*-Value | Chi-Square Ratio | 95-Confidence Intervals | |
|---|---|---|---|---|---|---|---|
| Lag 0 | | | | | | | |
| NO2_10ppb | -0.01558 | 0.01826 | 0.7282 | 0.3935 | 0.985 | 0.95 | 1.02 |
| AvgTemp | 0.00286 | 0.00181 | 2.4996 | 0.1139 | 1.003 | 0.999 | 1.006 |
| Lag 1 | | | | | | | |
| NO2_10ppb | 0.01325 | 0.01826 | 0.5271 | 0.4678 | 1.013 | 0.978 | 1.05 |
| AvgTemp | 0.00219 | 0.00182 | 1.445 | 0.2293 | 1.002 | 0.999 | 1.006 |
| Lag 2 | | | | | | | |
| NO2_10ppb | -0.00483 | 0.01826 | 0.0701 | 0.7912 | 0.995 | 0.96 | 1.031 |
| AvgTemp | 0.00215 | 0.00182 | 1.3991 | 0.2369 | 1.002 | 0.999 | 1.006 |
| Lag 3 | | | | | | | |
| NO2_10ppb | 0.02436 | 0.01822 | 1.7864 | 0.1814 | 1.025 | 0.989 | 1.062 |
| AvgTemp | 0.00296 | 0.00181 | 2.6654 | 0.1026 | 1.003 | 0.999 | 1.007 |
| Lag 4 | | | | | | | |
| NO2_10ppb | 0.01858 | 0.01825 | 1.0362 | 0.3087 | 1.019 | 0.983 | 1.056 |
| AvgTemp | 0.00057 | 0.00184 | 0.0967 | 0.7559 | 1.001 | 0.997 | 1.004 |
| Lag 5 | | | | | | | |
| NO2_10ppb | 0.03992 | 0.01839 | 4.7107 | 0.03 | 1.041 | 1.004 | 1.079 |
| AvgTemp | -0.00171 | 0.00186 | 0.8465 | 0.3576 | 0.998 | 0.995 | 1.002 |
| Lag 0-5 | | | | | | | |
| NO2_10ppb | 0.04196 | 0.03162 | 1.761 | 0.1845 | 1.043 | 0.98 | 1.11 |
| AvgTemp | 0.003 | 0.00254 | 1.3916 | 0.2381 | 1.003 | 0.998 | 1.008 |

Data were adjusted for apparent temperature (at same lag) analyses with a 7-day washout and 28-day referent periods. *Abbreviation Definitions: AvgTemp = Average temperature; Std Error=Standard Error.*

**Table S13.** Adults 18+: Asthma ED Visits for $SO_2$ (per 10 ppb). Single Pollutant, Single day lags and average lag.

| Parameter | Estimate | Std Error | Chi-Square | *p*-Value | Chi-Square Ratio | 95-Confidence Intervals | |
|---|---|---|---|---|---|---|---|
| Lag 0 | | | | | | | |
| SO2_10ppb | -0.00923 | 0.01553 | 0.3533 | 0.5522 | 0.991 | 0.961 | 1.021 |
| AvgTemp | 0.0026 | 0.00177 | 2.1423 | 0.1433 | 1.003 | 0.999 | 1.006 |
| Lag 1 | | | | | | | |
| SO2_10ppb | -0.00954 | 0.01559 | 0.3746 | 0.5405 | 0.991 | 0.961 | 1.021 |
| AvgTemp | 0.00249 | 0.00179 | 1.9471 | 0.1629 | 1.002 | 0.999 | 1.006 |
| Lag 2 | | | | | | | |
| SO2_10ppb | -0.0386 | 0.01606 | 5.7775 | 0.0162 | 0.962 | 0.932 | 0.993 |
| AvgTemp | 0.00223 | 0.00178 | 1.5629 | 0.2112 | 1.002 | 0.999 | 1.006 |
| Lag 3 | | | | | | | |
| SO2_10ppb | 0.01014 | 0.01557 | 0.4236 | 0.5151 | 1.01 | 0.98 | 1.042 |
| AvgTemp | 0.00335 | 0.00178 | 3.5164 | 0.0608 | 1.003 | 1 | 1.007 |
| Lag 4 | | | | | | | |
| SO2_10ppb | -0.00623 | 0.01529 | 0.166 | 0.6837 | 0.994 | 0.964 | 1.024 |
| AvgTemp | 0.00096 | 0.0018 | 0.2896 | 0.5905 | 1.001 | 0.997 | 1.005 |
| Lag 5 | | | | | | | |
| SO2_10ppb | 0.00287 | 0.01542 | 0.0348 | 0.8521 | 1.003 | 0.973 | 1.034 |
| AvgTemp | -0.00093 | 0.00183 | 0.2593 | 0.6106 | 0.999 | 0.995 | 1.003 |
| Lag 0-5 | | | | | | | |
| SO2_10ppb | -0.03014 | 0.03035 | 0.9862 | 0.3207 | 0.97 | 0.914 | 1.03 |
| AvgTemp | 0.00356 | 0.00251 | 2.0007 | 0.1572 | 1.004 | 0.999 | 1.009 |

Data were adjusted for apparent temperature (at same lag) analyses with a 7-day washout and 28-day referent periods. *Abbreviation Definitions: AvgTemp = Average temperature; Std Error=Standard Error.*

**Table S14.** Adults 18+: Asthma ED Visits for $PM_{2.5}$ (per 10 μg/$m^3$), Ozone (per 10 ppb), CO (ppm), NO2 (per 10 ppb), SO2 (per 10 ppb). Multiple Pollutant models, Single day lags and average lag.

| Parameter | Estimate | Std Error | Chi-Square | *p*-Value | Chi-Square Ratio | 95-Confidence Intervals | |
|---|---|---|---|---|---|---|---|
| Lag 0 | | | | | | | |
| PM2.5_10μg | 0.02193 | 0.0359 | 0.3732 | 0.5412 | 1.022 | 0.953 | 1.097 |
| O3_10ppb | -0.03005 | 0.0168 | 3.1968 | 0.0738 | 0.97 | 0.939 | 1.003 |
| NO2_10ppb | -0.03612 | 0.02325 | 2.4141 | 0.1202 | 0.965 | 0.922 | 1.009 |
| SO2_10ppb | -0.00864 | 0.01581 | 0.2991 | 0.5844 | 0.991 | 0.961 | 1.023 |
| CO_ppm | 0.13596 | 0.07659 | 3.1513 | 0.0759 | 1.146 | 0.986 | 1.331 |
| AvgTemp | 0.00305 | 0.00193 | 2.498 | 0.114 | 1.003 | 0.999 | 1.007 |
| Lag 1 | | | | | | | |
| PM2.5_10μg | -0.00576 | 0.03564 | 0.0261 | 0.8715 | 0.994 | 0.927 | 1.066 |
| O3_10ppb | -0.03319 | 0.01682 | 3.8911 | 0.0485 | 0.967 | 0.936 | 1 |
| NO2_10ppb | 0.01498 | 0.02312 | 0.4194 | 0.5172 | 1.015 | 0.97 | 1.062 |
| SO2_10ppb | -0.0108 | 0.01586 | 0.4636 | 0.4959 | 0.989 | 0.959 | 1.02 |
| CO_ppm | 0.05448 | 0.07603 | 0.5134 | 0.4737 | 1.056 | 0.91 | 1.226 |
| AvgTemp | 0.00309 | 0.00194 | 2.5427 | 0.1108 | 1.003 | 0.999 | 1.007 |
| Lag 2 | | | | | | | |
| PM2.5_10μg | -0.00281 | 0.03612 | 0.006 | 0.938 | 0.997 | 0.929 | 1.07 |
| O3_10ppb | -0.04036 | 0.01681 | 5.7604 | 0.0164 | 0.96 | 0.929 | 0.993 |
| NO2_10ppb | 0.00157 | 0.02297 | 0.0047 | 0.9455 | 1.002 | 0.957 | 1.048 |
| SO2_10ppb | -0.03851 | 0.01629 | 5.5865 | 0.0181 | 0.962 | 0.932 | 0.993 |
| CO_ppm | 0.05366 | 0.07731 | 0.4817 | 0.4877 | 1.055 | 0.907 | 1.228 |
| AvgTemp | 0.00336 | 0.00194 | 2.9991 | 0.0833 | 1.003 | 1 | 1.007 |
| Lag 3 | | | | | | | |
| PM2.5_10μg | -0.03443 | 0.0364 | 0.8946 | 0.3442 | 0.966 | 0.9 | 1.038 |
| O3_10ppb | -0.03373 | 0.01669 | 4.0833 | 0.0433 | 0.967 | 0.936 | 0.999 |
| NO2_10ppb | 0.02868 | 0.02308 | 1.5442 | 0.214 | 1.029 | 0.984 | 1.077 |
| SO2_10ppb | 0.00924 | 0.01585 | 0.3399 | 0.5599 | 1.009 | 0.978 | 1.041 |
| CO_ppm | 0.06849 | 0.07721 | 0.7869 | 0.375 | 1.071 | 0.921 | 1.246 |
| AvgTemp | 0.0042 | 0.00193 | 4.7154 | 0.0299 | 1.004 | 1 | 1.008 |
| Lag 4 | | | | | | | |
| PM2.5_10μg | 0.0117 | 0.03693 | 0.1003 | 0.7515 | 1.012 | 0.941 | 1.088 |
| O3_10ppb | -0.03637 | 0.01655 | 4.8275 | 0.028 | 0.964 | 0.934 | 0.996 |
| NO2_10ppb | 0.02858 | 0.02297 | 1.5483 | 0.2134 | 1.029 | 0.984 | 1.076 |
| SO2_10ppb | -0.00775 | 0.01554 | 0.2486 | 0.6181 | 0.992 | 0.963 | 1.023 |
| CO | ppm | -0.04151 | 0.0761 | 0.2976 | 0.5854 | 0.959 | 0.826 |
| AvgTemp | 0.00179 | 0.00196 | 0.8412 | 0.359 | 1.002 | 0.998 | 1.006 |
| Lag 5 | | | | | | | |
| PM2.5_10μg | -0.00387 | 0.03728 | 0.0108 | 0.9173 | 0.996 | 0.926 | 1.072 |
| O3_10ppb | -0.02249 | 0.01654 | 1.8495 | 0.1738 | 0.978 | 0.947 | 1.01 |
| NO2_10ppb | 0.03416 | 0.02321 | 2.1676 | 0.1409 | 1.035 | 0.989 | 1.083 |
| SO2_10ppb | -0.00142 | 0.01575 | 0.0082 | 0.928 | 0.999 | 0.968 | 1.03 |
| CO_ppm | 0.07339 | 0.07517 | 0.9533 | 0.3289 | 1.076 | 0.929 | 1.247 |
| AvgTemp | -0.00113 | 0.00199 | 0.323 | 0.5698 | 0.999 | 0.995 | 1.003 |
| Lag 0-5 | | | | | | | |

| | | | | | | | |
|---|---|---|---|---|---|---|---|
| PM2.5_10μg | 0.00246 | 0.06333 | 0.0015 | 0.969 | 1.002 | 0.885 | 1.135 |
| O3_10ppb | -0.0968 | 0.02892 | 11.2029 | 0.0008 | 0.908 | 0.858 | 0.961 |
| NO2_10ppb | 0.04295 | 0.04435 | 0.9381 | 0.3328 | 1.044 | 0.957 | 1.139 |
| SO2_10ppb | -0.03956 | 0.03091 | 1.6375 | 0.2007 | 0.961 | 0.905 | 1.021 |
| CO_ppm | 0.14951 | 0.13189 | 1.285 | 0.257 | 1.161 | 0.897 | 1.504 |
| AvgTemp | 0.00572 | 0.00273 | 4.4001 | 0.0359 | 1.006 | 1 | 1.011 |

Data were adjusted for apparent temperature (at same lag) analyses with a 7-day washout and 28-day referent periods. *Abbreviation Definitions: AvgTemp = Average temperature; Std Error=Standard Error.*
